# Metastatic Squamous Cell Carcinoma of the Lung Disclosed From Constipation Workup

**DOI:** 10.14309/crj.0000000000001133

**Published:** 2023-08-30

**Authors:** Zilan X. Lin, Aaron Weiss, Kyu-In Lee, Gabriel Heering, Lillian Chang, Shireen Pais

**Affiliations:** 1 Division of Gastroenterology and Hepatobiliary Diseases, Westchester Medical Center, Valhalla, NY; 2 Department of Medicine, Westchester Medical Center, Valhalla, NY

**Keywords:** metastatic lung cancer, rectal mass, colorectal metastasis, constipation

## Abstract

A palpable rectal mass associated with gastrointestinal (GI) symptoms immediately raises concern for colorectal cancer, but rarely can represent distant metastatic disease. The incidence of symptomatic colorectal metastasis from a primary lung cancer without any pulmonary symptom is extremely rare. We report a rare case of constipation as the presenting symptom in a patient ultimately found to have metastatic squamous cell carcinoma of the lung. A rectal mass was readily palpable on examination, illustrating the importance of digital rectal examination. In addition, GI clinicians should maintain a high index of suspicion when evaluating patients at risk of non-GI malignancies.

## INTRODUCTION

Lung cancer is extremely common, occurring in approximately 2.2 million patients in 2020. While the number of deaths is declining because of decreased smoking among the population, lung cancer remains the leading cause of cancer-associated death in the United States.^1,2^ Squamous cell carcinoma of the lung typically presents with respiratory symptoms and frequently metastasizes to the bone, liver, adrenal glands, and brain.^3–5^ However, gastrointestinal (GI) metastases, especially involving distal sites, such as the colon and rectum, are extremely unusual. Symptomatic GI metastases are even more uncommon. We report a rare case of constipation as the presenting symptom that led to the detection of a widely metastatic squamous cell lung carcinoma with rectal involvement.

## CASE REPORT

A 67-year-old man was referred to the gastroenterology clinic from an outside hospital for progressively worsening constipation and incomplete stool evacuation for 3 months. He had no significant medical history, except a 30 pack-year history of smoking. He also had a family history notable for colon cancer in his father (died of the disease at 62 years) as well as 2 other family members with breast and ovarian cancers. He had no pulmonary symptoms or other complaints. His rectal examination was remarkable for a firm mass 6 cm from the anal verge. The patient denied any other concerning symptoms, such as nausea, vomiting, weight loss, anorexia, melena, or hematochezia, and a colonoscopy 5 years earlier was only notable for a colonic polyp. A computed tomography scan obtained a few days before the initial office visit revealed right rectal wall thickening.

**Figure 1.**
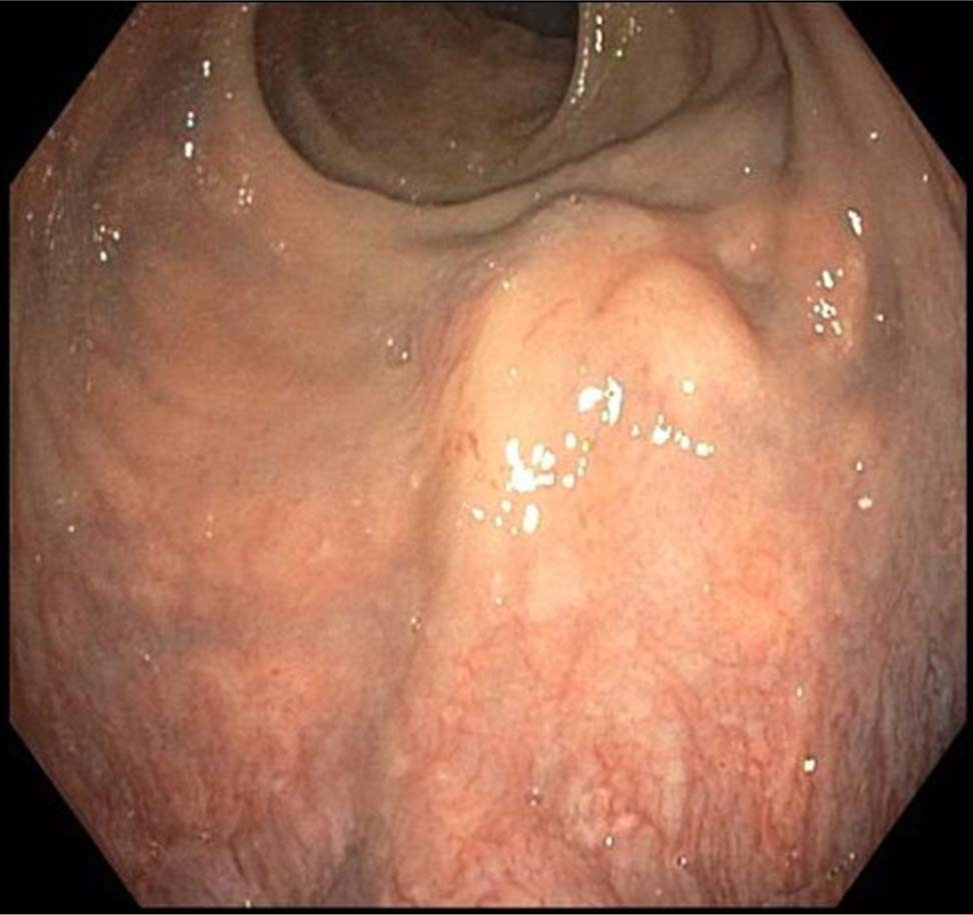
Endoscopic view of the submucosal rectal mass.

**Figure 2.**
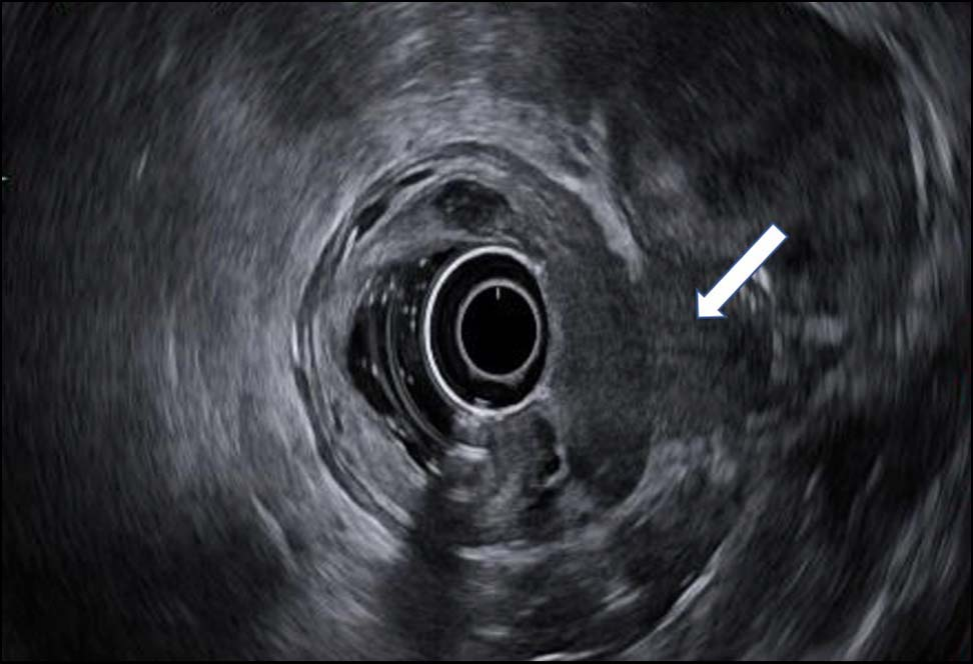
Mass (white arrow) abutting the rectum and prostate on endoscopic ultrasound.

**Figure 3.**
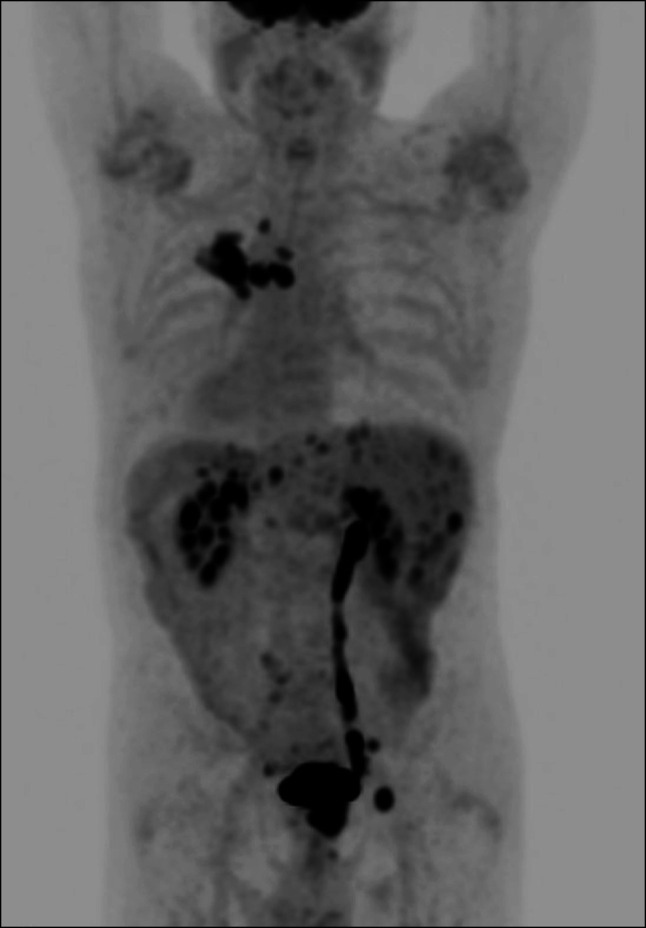
Positron emission tomography scan showing extensive pulmonary and liver disease.

The patient's presentation was initially concerning for rectal cancer, possibly hereditary nonpolyposis colorectal cancer given his extensive family history. He underwent an urgent colonoscopy, which showed a subepithelial mass in the distal rectum (Figure 1). An extrinsic 32 mm mass was identified on an endoscopic ultrasound abutting the prostate and rectum (Figure 2). Fine-needle biopsy was positive for squamous cell carcinoma. A subsequent positron emission tomography scan showed a large left perihilar lung mass with associated lymphadenopathy as well as metastatic disease to the liver, acetabulum, prostate, and rectum (Figure 3). The findings were communicated with the local referring doctor for further management and genetic testing, and the patient was referred to oncology at an outside hospital for evaluation for palliative treatment with chemotherapy and/or radiation.

## DISCUSSION

A rectal mass associated with solitary GI symptoms is highly suspicious for a primary colorectal cancer. However, in our case, further investigation revealed metastatic cancer originating from the lung. Metastatic neoplasm in the colon is exceedingly rare, comprising less than 1% of all colorectal cancers, and typically is diagnosed in patients with a known primary malignancy, most commonly ovarian, lung, breast, prostate, kidney, or melanoma.^6^ Meanwhile, primary pulmonary malignancies, which are found to have metastases at initial diagnosis in 50% of cases, seldom spread to the GI system (they do commonly metastasize to the mediastinum, lymph nodes, adrenals, liver, bone, and brain).^3–5^ Yang et al reviewed all cases of lung cancer from January 2003 to April 2005 at a tertiary-care Taiwanese hospital and found 6 of 339 patients (1.77%) with primary lung cancer demonstrating symptomatic GI metastasis.^7^ Furthermore, there is variation in rates of GI metastasis by location, with distal GI metastases being far less common. In a retrospective study, Min Soo Kim et al showed 10 of 5,239 patients with lung cancer (0.19%) had GI metastases and half of those patients had small bowel metastases; in addition, only 2 patients had metastases to the colon.^8^ No previously published cases to our knowledge have discussed metastatic lung cancer to the rectum specifically.

A well-known, widely studied independent risk factor of lung cancer is tobacco smoking. Our patient had significant smoking history, which might be the only clue pointing to the diagnosis of the primary lung cancer. However, the health effects of smoking are broad, and smoking can lead to cardiovascular diseases, respiratory issues, musculoskeletal problems, and cancers.^9^ Therefore, in the current case, smoking history alone would not necessarily elicit any different workup.

Despite the rarity, clinicians should maintain a suspicion for a pulmonary malignancy metastasizing to the colon because, regardless of symptoms, such cases will typically carry a poor prognosis with a mean survival of only 2–3 months.^3,10–12^ In fact, in some cases, the colon metastases are found only on autopsy.^11^ Most commonly, these patients will be found to have pulmonary malignancy after they develop respiratory symptoms, such as exertional dyspnea and hemoptysis. Only later will GI symptoms manifest and their intestinal involvement become evident.^5,8,13^ One case discussed patients afflicted with pulmonary malignancies who developed respiratory symptoms shortly after initially presenting with GI symptoms.^3^ Patients diagnosed with metastatic pulmonary cancer without any respiratory symptoms, as in our case, are extremely rare and not previously reported in the literature, to our best knowledge.

Various GI symptoms have been described in patients with metastatic lung cancer. Abdominal pain and anemia are the most common presenting symptoms.^11^ Other symptoms include diarrhea, bowel obstruction, perforation, fistula, and occult or frank GI bleeding.^12,14–18^ In our case, the patient appeared to be otherwise healthy, with no pulmonary symptoms and constipation as his only complaint despite advanced metastatic lung cancer.

On presentation to the GI clinic, our patient was immediately evaluated with a rectal examination, which demonstrated the mass. When a Blumer shelf (tumor metastasized to the rectouterine pouch, or pouch of Douglas) is palpated on examination, malignancy (classically gastric cancer) should be considered. However, other malignances occur as well, and lung, pancreas, gastric, and ovarian cancers have been reported in association with a Blumer shelf.^19,20^

Our case is notable for its extreme rarity. It also illustrates the importance of maintaining an appropriate index of suspicion when evaluating patients at risk of non-GI malignancies and the indispensability of the digital rectal examination when evaluating patients with rectal symptoms.

## DISCLOSURES

Author contributions: ZX Lin wrote the manuscript. A. Weiss, K. Lee, G. Heering, L. Chang, and S. Pais revised the manuscript. S. Pais is the article guarantor. All authors approved the final manuscript.

Financial disclosure: None to report.

Previous presentation: This case was presented at the 2020 Virtual Annual Meeting of the American College of Gastroenterology; October 2020.

Informed consent could not be obtained for this case report. All identifying information have been removed.
